# Nipple–Areola Complex Reconstruction Using FixNip NRI Implant after Mastectomy: An Innovative Technique

**DOI:** 10.1007/s00266-024-04418-y

**Published:** 2024-10-04

**Authors:** Serena Iacovelli, Giuseppe De Palma, Valerio De Santis, Daniela Anna Cutrignelli, Andrea Armenio, Samantha Bove, Maria Colomba Comes, Annarita Fanizzi, Elsa Vitale, Raffaella Massafra, Cosmo Maurizio Ressa

**Affiliations:** 1 Scientific Directorate, IRCCS Istituto Tumori “Giovanni Paolo II”, Bari, Italy; 2 Institutional BioBank, Experimental Oncology and Biobank Management Unit, IRCCS Istituto Tumori “Giovanni Paolo II”, Bari, Italy; 3 Plastic and Reconstructive Surgery Unit, IRCCS Istituto Tumori “Giovanni Paolo II”, Bari, Italy; 4 Biostatistics and Bioinformatic Lab, IRCCS Istituto Tumori “Giovanni Paolo II”, Bari, Italy

**Keywords:** Areola, Mastectomy, Reconstruction, Nipple, FixNip

## Abstract

**Background:**

Nipple–areolar complex reconstruction is the final stage of breast reconstruction, and it improves quality of life in patients with post-mastectomy breast cancer. We present a case of a patient with breast cancer underwent breast reconstruction and subsequent nipple–areolar complex reconstruction with an innovative biocompatible smooth silicone implant specially designed for a long-lasting restoration of the nipple–areola complex called FixNip NRI. However, to our knowledge, nipple–areolar complex reconstruction with FixNip was not previously reported.

**Innovative Technique:**

We present an emerging technique applied on a patient with breast cancer treated with skin-sparing mastectomy and with immediate breast reconstruction using an expander and then exchanged expander to breast implant. FixNip nipple reconstruction implant is implanted for the reconstruction of the areola–nipple complex with local–regional anaesthesia. She did not develop any postoperatively short-term or long-term complications, and her nipple slowly underwent to a gradual and better definition of its profile.

**Conclusion:**

This new approach regarding the reconstruction of the nipple–areola complex seems to be very promising in relation to both the degree of aesthetic satisfaction of patients and the ease of use by surgeons.

**Level of Evidence V:**

This journal requires that authors assign a level of evidence to each article. For a full description of these Evidence-Based Medicine ratings, please refer to the Table of Contents or the online Instructions to Authors www.springer.com/00266.

## The Case

**Fig. 1:**
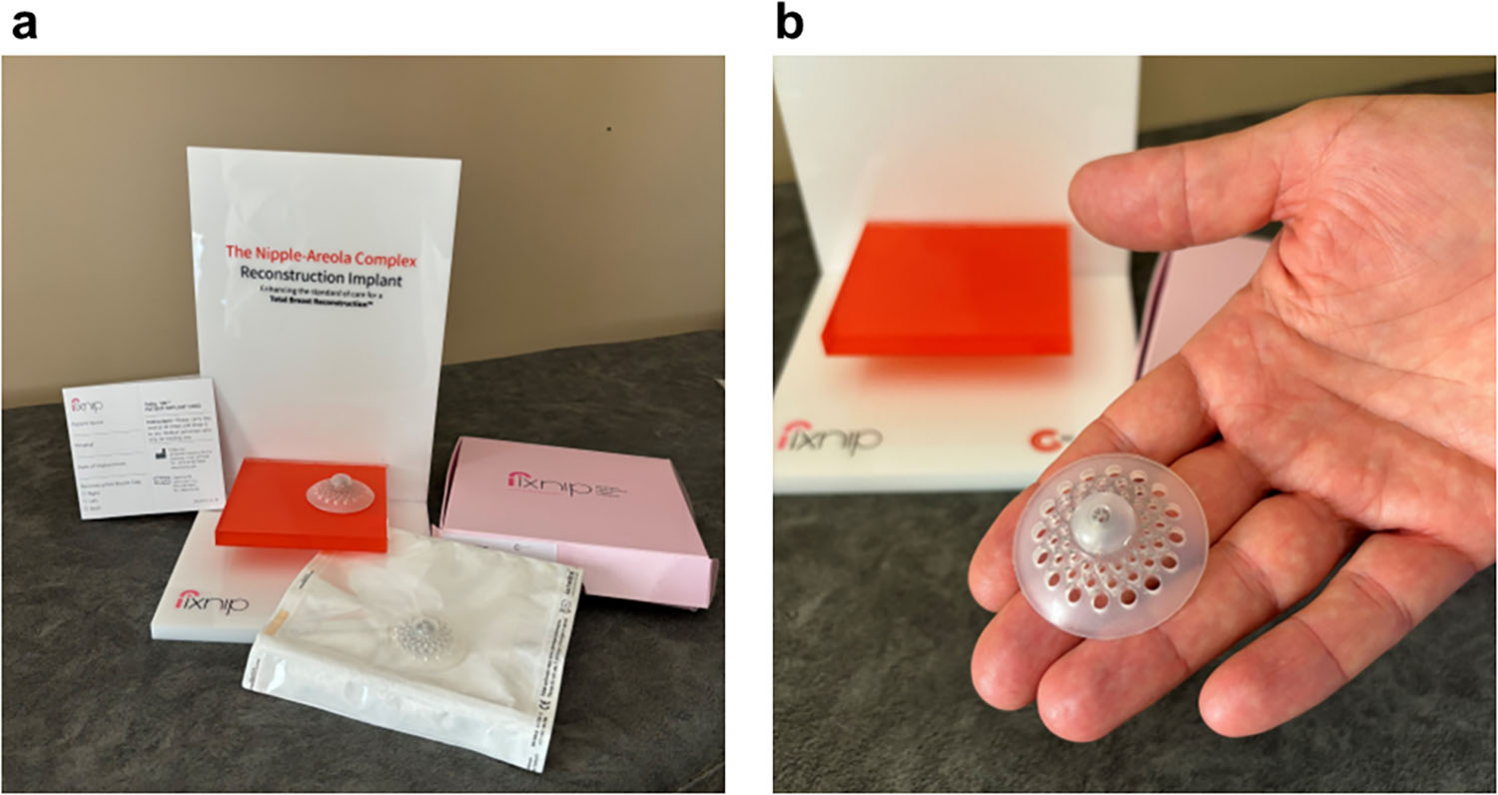
**a** View of FixNip NRI box and which is included inside and **b** view of FixNip NRI

We report the case of a 59-year-old patient who was diagnosed with right breast carcinoma. She was a smoker, and she had history of hypertension, with BRCA1 and BRCA2 mutated. She underwent right breast mastectomy and lymphectomy with immediate reconstruction with expander in May 2019 and on drug therapy with letrozole. In the absence of any obvious complications, in June 2022, the patient was operated again for second reconstructive surgical step for expander replacement with prosthesis, and in October of the same year, after ultrasound evaluation of the breast soft tissues to allow adequate prosthetic implant thickness, she was implanted with the innovative FixNip nipple reconstruction implant (NRI) (Fixnip Ltd., Caesarea, Israel) for reconstruction of the nipple–areolar complex (NAC). The implant includes a nitinol frame designed to provide mechanical structure that is fully covered by the silicone and has no contact with the breast tissue (Fig. 1). The implant provides the physician with a stable platform for the nipple structure.

As reported in the medical record, the patient reports a physiologic history of childbirth and a remote pathologic history of adolescent hepatitis and denies allergies.

The first surgery the patient underwent in May 2019 was a skin-sparing mastectomy to the right breast and immediate reconstruction with a 550 cc breast expander, with an initial volume of 80 cc and placed below the pectoralis major muscle and dentate muscle fascia. Subsequently during follow-ups lasting about 1 year, progressive expansion was achieved to a volume of 450 on 550 cc of physiological saline solution.

In June 2022, the patient underwent expander replacement surgery with a right breast prosthesis, partial capsulectomy, reshaping of the prosthetic pocket to improve the housing of the definitive implant and insertion of 390 cc prosthesis (Mentor Medical Systems, Lieden, Netherlands). The postoperative course was free of complications and smooth in its progress. The time lapse between the insertion of the expander and the subsequent replacement with the definitive prosthesis was motivated firstly by the slow and gradual expansions, 30–50 cc expansion every 30 days, in turn also justified by the lymphectomy performed so as to minimize the complications of lymphedema or seroma formation and which allowed constant follow-up of the patient. Secondly, long waiting time in the Italian National Health Service that is publicly funded. Furthermore, this was affected by the patient’s initial discomfort in taking letrozole therapy which added further delay.

**Fig. 2:**
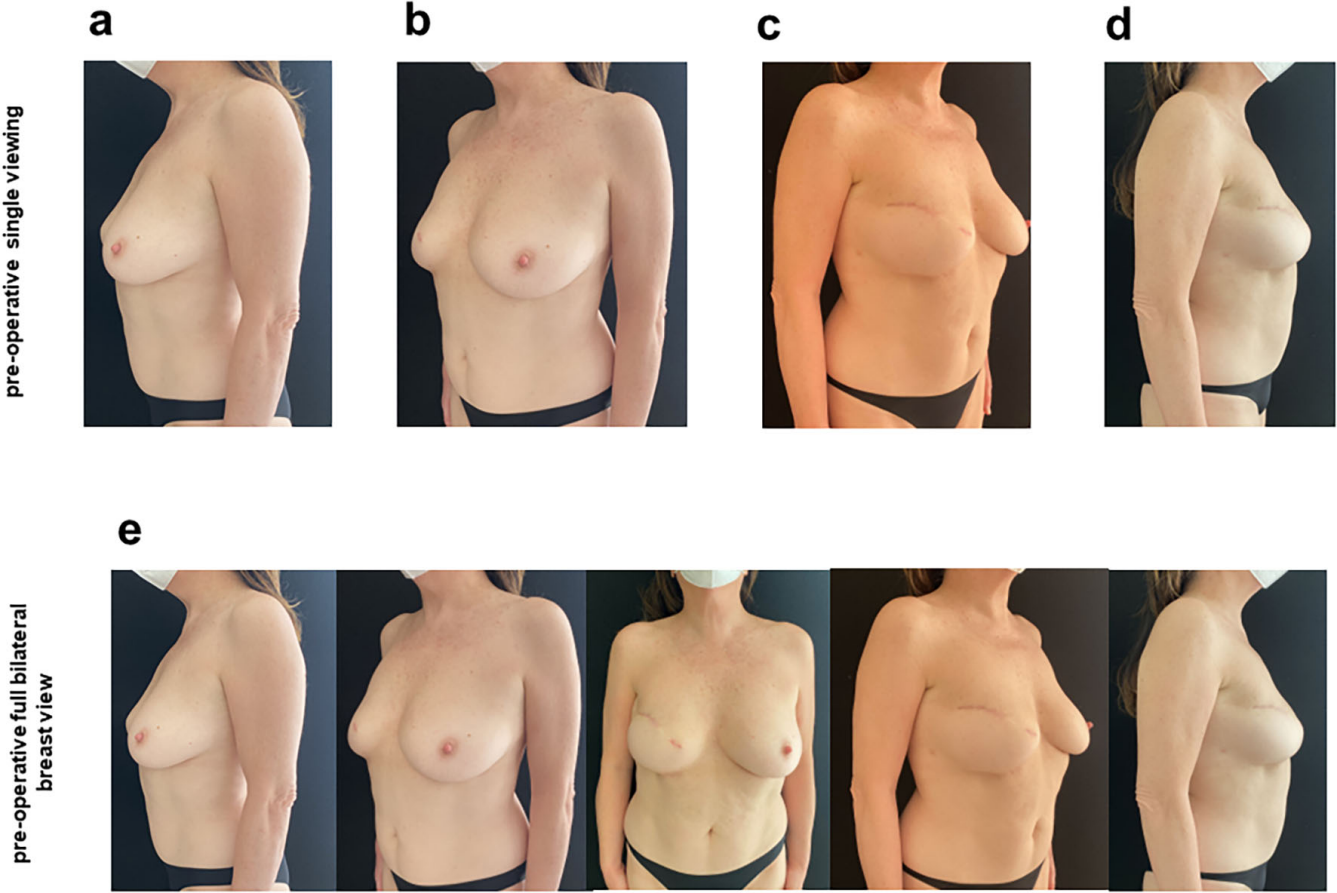
Pre-operative photographs about the FixNip prosthesis implantation **a** lateral left breast view, **b** oblique 45-degree left view, **c** lateral right breast view, **d** oblique 45-degree right view and **e** full bilateral breast view

The nipple prosthesis insertion surgery for nipple reconstruction, performed in October 2022 in the day service outpatient clinic of the Plastic and Reconstructive Surgery Unit of the IRCCS Istituto Tumori “Giovanni Paolo II” in Bari, with loco-regional anesthesia, lasted 30 min **(**Fig. 2). The 4-month time between the application of the breast implant and the nipple prosthesis was useful for the periprosthetic capsule formation around implant, without exposing the breast implant and to avoid the occurrence of complications such as bacterial and fungal superinfections.

**Fig. 3:**
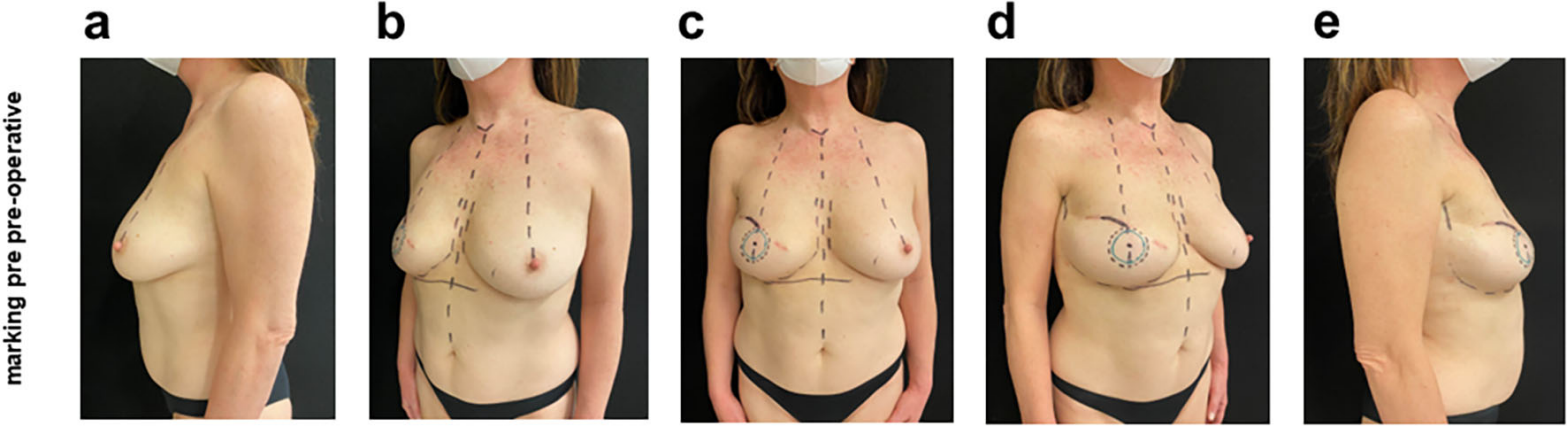
Pre-operative marking of the FixNip position **a** Morking pre-op lato sx, **b** Morking pre-op ¾ sx, **c** Morking pre-op frontal view, **d** Morking pre-op 3/4 lato dx and **e** Morking pre-op lato dx

**Fig. 4:**
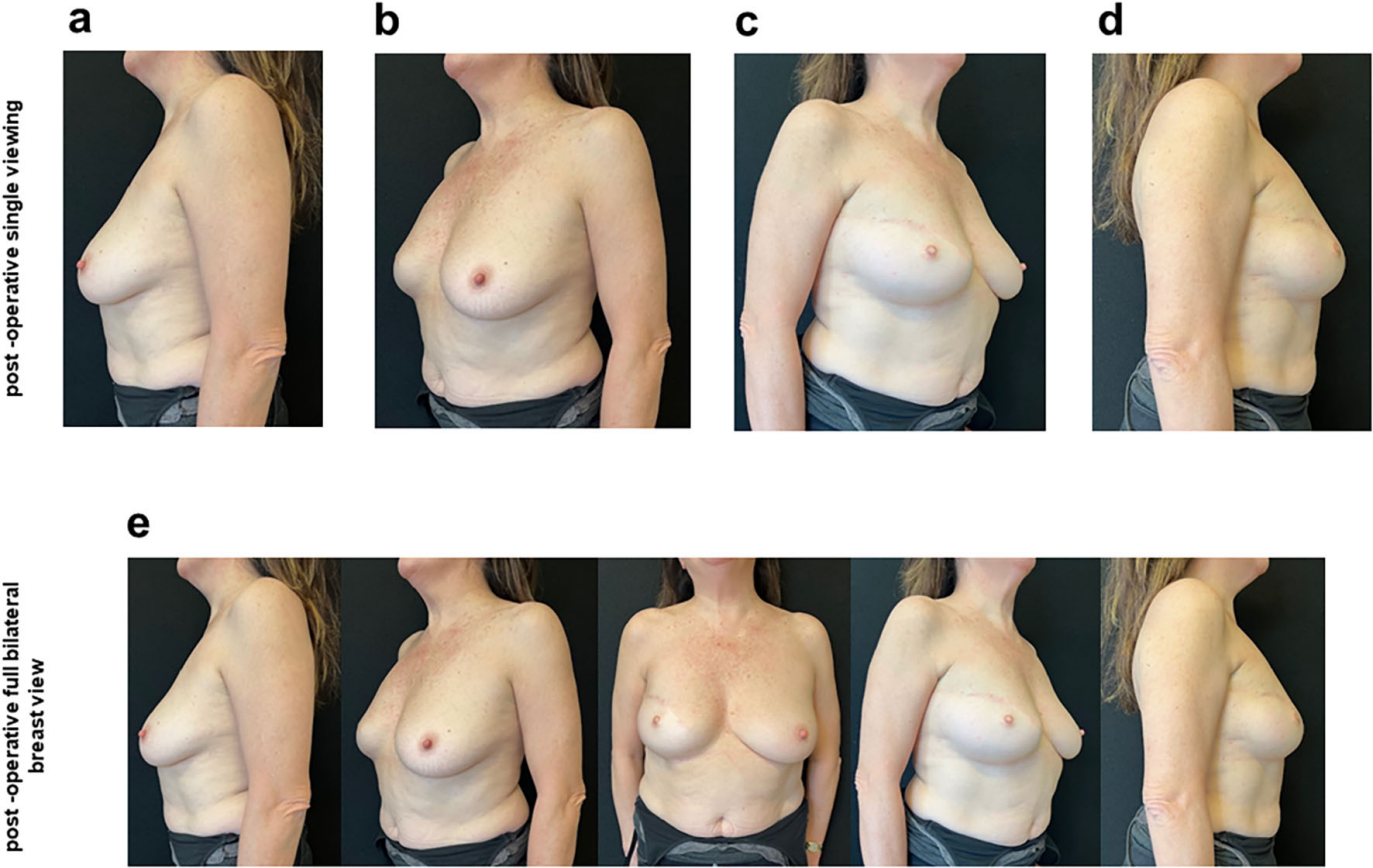
Photographs at 12 months postoperative FixNip prosthesis implantation **a** lateral left breast view, **b** oblique 45-degree left view, **c** lateral right breast view, **d** oblique 45-degree right view and **e** full bilateral breast view

The surgical procedure performed for NAC implantation included a pre-operative ultrasound study for thorough evaluation of the subcutaneous soft tissues in the right breast, a method performed with a 4–15 MHz linear probe. A soft tissue thickness greater than 2 cm was found. A marking pre-surgery was performed before the surgical procedure (Fig. 3). Therefore, excision of previous skin scar, soft tissue dieresis, establishment of subcutaneous/hypodermic pocket, washing of the pocket with saline and betadine, insertion of 45 mm diameter FixNip NRI, layered suture and finally compressive dressing with exposure of the neo-nipple were performed. The patient returned to outpatient visit with weekly recurrence in the 1st month and then after 1, 3, 6, 9 and 12 months. The patient performed a postoperative course and serious outpatient follow-ups with dedicated dressings. No short- or long-term complications or noteworthy items were reported (Fig. 4).

## Clinical Findings

During follow-up, in the first 30 days after NAC prosthesis application, a change in the skin overlying the nipple was seen. Initially white in colour with slightly raised skin and a poorly defined nipple, later in follow-ups from 15 days onwards, a reddening of the skin with hyper-projection of the nipple and a better definition of the morphology with thickening of the skin were appreciated. The patient reported no short- or long-term complications. She reported no signs of pain or inflammation except slightly reddened skin in the central of NAC portion until 6 months after surgery. On ultrasonographic evaluation at 6 months of the implant region, we reported the implant in place with fibrous capsule formation and fibrous bridges between the anterior and posterior surfaces of the device. Postoperatively, the patient performed antibiotic prophylaxis with amoxicillin combined with clavulanic acid.

## Discussion

Breast cancer treatment includes a total or partial mastectomy followed by breast reconstruction. The latter represents a series of clinical, surgical and psychological processes that involve patients in every aspect of the emotional and health sphere. If at the time of diagnosis, the main concern for patients concerns the presence of the tumour and therefore the success of the demolition surgery, subsequently the psychological aspect prevails due to the impairment suffered.

To conclude a path of illness and rebirth, over the years, there has been an ever-increasing demand to be able to conclude this process through the NAC reconstruction.

An innovative NAC prosthesis called FixNip NRI has been developed for aesthetic improvement of the female nipple with softer feel and natural appearance. It is an implantable solid hypodermic implant intended to be placed under the skin. FixNip prototype has been presented in 2017, and then, the first surgery was on October 2019. Some authors have reported the potential of FixNip NRI despite not having had direct clinical experience with their implantation and suggested that clinical results and complication rates on patients should be verified over time [1].

Herein, we report our experience of treating a patient with breast cancer underwent breast reconstruction and subsequent NAC reconstruction with the FixNip NRI with aesthetically satisfactory outcomes.

After the first mastectomy surgery, plastic surgeons perform reconstruction, which can take place at one time since the mastectomy or later, at a later time. At the end of the reconstruction with prosthetic implant or expander, to date, it is possible to reconstruct the NAC, which allows to give back to the woman an important part related to femininity. Although various techniques for nipple reconstruction are described in the literature, the most challenging aspect of NAC reconstruction is maintenance of a long-lasting projection. Materials for NAC reconstruction to achieve this goal can be obtained from autologous tissues as dermis [2, 3], adipose tissue [4] and cartilage (from the ribs or the outer ear) [5]. However, utilizing auricular tissues resulted in unnatural contour and produced donor site scars. NAC prostheses represent a strong point for all those women facing a clinically and physically important path. The use of the FixNip NRI allows, during the healing process of the wounds and the formation of the periprosthetic capsule, fibrotic strands to form in the holes present in the periphery of the prosthesis, highlighting the nipple in a permanent and more lasting manner, unlike reconstruction with autologous tissues, which over time inevitably leads to a flattening of the nipple. The advantage of FixNip NRI is that with one implantation the nipple can be permanently restored, unlike other nipple prosthesis, such as Naturally Impressive LLC, that last for an average of 2 weeks per application.

Although the innovativeness and recent development of these implants does not yet allow the complications associated with them to be delineated with great statistics, clinicians tend to relate them to complications of implants commonly used for breast reconstruction and may include: allergic reactions, inflammation, implant migration, implant extrusion/exposure, interaction with underlying breast implant, seroma, haematoma, numbness, paraesthesias, infection, tissue ischaemia, localized necrosis, epidermolysis, skin discoloration, hardening, flattening, implant exposure, bleeding, insufficient or excessive augmentation, pressure sores, skin breakdown, capsular contracture, implant inversion (flipping), implant folding, unnatural discomfort/sensation and pain. Finally, not to be underestimated is the degree of patient satisfaction. FixNip NRIs are a definitive aesthetic alternative to support soft tissue where it is weak, offering durable projection and allowing reconstruction of the areola–nipple complex during the final surgical phase of breast reconstruction [6]. Also to be evaluated among the possible consequences are post-tattoo complications that surgeons suggest 3–6 months after FixNip NRI implantation just as it can very often be useful to also touch-up the contralateral nipple to obtain the best possible colour match.
