# Correction: Nipple–Areola Complex Reconstruction Using FixNip NRI Implant after Mastectomy: An Innovative Technique

**DOI:** 10.1007/s00266-024-04492-2

**Published:** 2024-10-30

**Authors:** Serena Iacovelli, Giuseppe De Palma, Valerio De Santis, Daniela Anna Cutrignelli, Andrea Armenio, Samantha Bove, Maria Colomba Comes, Annarita Fanizzi, Elsa Vitale, Raffaella Massafra, Cosmo Maurizio Ressa

**Affiliations:** 1 Scientific Directorate, IRCCS Istituto Tumori “Giovanni Paolo II”, Bari, Italy; 2 Institutional BioBank, Experimental Oncology and Biobank Management Unit, IRCCS Istituto Tumori “Giovanni Paolo II”, Bari, Italy; 3 Plastic and Reconstructive Surgery Unit, IRCCS Istituto Tumori “Giovanni Paolo II”, Bari, Italy; 4 Biostatistics and Bioinformatic Lab, IRCCS Istituto Tumori “Giovanni Paolo II”, Bari, Italy

**Correction to: Aesthetic Plastic Surgery** 10.1007/s00266-024-04418-y

The original online version of this article was revised to correct funding information. The correct funding information for this article is as follows:

This research received “Ricerca Corrente 2024” funding from the Italian Ministry of Health to cover publication costs.

The original article has been corrected.

